# Effects of Wind Speed on Size-Dependent Morphology and Composition of Sea Spray Aerosols

**DOI:** 10.1021/acsearthspacechem.4c00119

**Published:** 2024-07-16

**Authors:** Chamika K. Madawala, Carolina Molina, Deborah Kim, Dilini Kirindigoda Gamage, Mengnan Sun, Raymond J. Leibensperger, Lincoln Mehndiratta, Jennie Lee, Chathuri P. Kaluarachchi, Ke’La A. Kimble, Greg Sandstrom, Charbel Harb, Julie Dinasquet, Francesca Malfatti, Kimberly A. Prather, Grant B. Deane, M. Dale Stokes, Christopher Lee, Jonathan H. Slade, Elizabeth A. Stone, Vicki H. Grassian, Alexei V. Tivanski

**Affiliations:** † Department of Chemistry, University of Iowa, Iowa City, Iowa 52242, United States; ‡ Department of Chemistry and Biochemistry, University of California San Diego, La Jolla, California 92093, United States; § Scripps Institution of Oceanography, University of California San Diego, La Jolla, California 92093, United States; ∥ Department of Life Science, Universita’ degli Studi di Trieste, Trieste 34127, Italy

**Keywords:** sea spray aerosol, atomic force microscopy, wind speed, morphology, composition, single particle

## Abstract

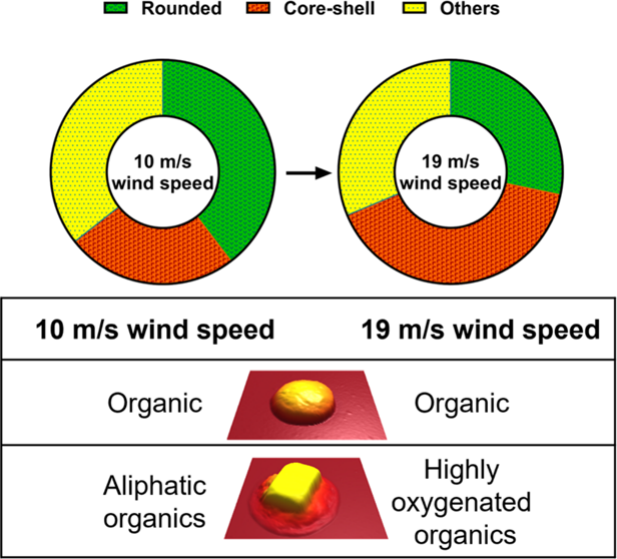

Variable wind speeds over the ocean can have a significant impact on the formation mechanism and physical-chemical properties of sea spray aerosols (SSA), which in turn influence their climate-relevant impacts. Herein, for the first time, we investigate the effects of wind speed on size-dependent morphology and composition of individual nascent SSA generated from wind-wave interactions of natural seawater within a wind-wave channel as a function of size and their particle-to-particle variability. Filter-based thermal optical analysis, atomic force microscopy (AFM), AFM infrared spectroscopy (AFM-IR), and scanning electron microscopy (SEM) were employed in this regard. This study focuses on SSA with sizes within 0.04–1.8 μm generated at two wind speeds: 10 m/s, representing a wind lull scenario over the ocean, and 19 m/s, indicative of the wind speeds encountered in stormy conditions. Filter-based measurements revealed a reduction of the organic mass fraction as the wind speed increases. AFM imaging at 20% relative humidity of individual SSA identified six main morphologies: prism-like, rounded, core–shell, rod, rod inclusion core–shell, and aggregates. At 10 m/s, most SSA were rounded, while at 19 m/s, core–shells became predominant. Based on AFM-IR, rounded SSA at both wind speeds had similar composition, mainly composed of aliphatic and oxygenated species, whereas the shells of core–shells displayed more oxygenated organics at 19 m/s and more aliphatic organics at 10 m/s. Collectively, our observations can be attributed to the disruption of the sea surface microlayer film structure at higher wind speeds. The findings reveal a significant impact of wind speed on morphology and composition of SSA, which should be accounted for accurate assessment of their climate effects.

## Introduction

Sea spray aerosols (SSA) are one of the most abundant types of natural atmospheric aerosols that accounts for a significant contribution to the total aerosol mass concentration in the atmosphere.^1^ SSA are generated upon the bursting of air bubbles entrained in the ocean from breaking waves through wind-driven mechanisms.^1−6^ Once airborne, SSA have significant impact on Earth’s radiative budget directly by scattering and absorbing solar light, or indirectly by acting as cloud condensation nuclei (CCN) or ice nucleating particles.^4,7−12^ During bubble bursting, the organic, inorganic, and biological species in seawater and at sea surface microlayer (SML, the uppermost layer with submicrometer thickness, which is enriched in organics relative to underlying seawater) can be transferred into SSA.^13−17^ SSA can be produced via bubble-cap bursting (i.e., film drops) which are enriched with organic matter or via bubble-cavity collapse (i.e., jet drops), which are predominantly inorganic salts.^18^ Moreover, the SML film structure and composition can modulate the composition and physical-chemical properties of SSA produced via film drops.^18,19^ Because of the complex chemical nature of SML, SSA are highly complex size-dependent mixtures of many chemical and biological species at various morphologies and mixing states.^18,20−25^ Under high winds (>9 m/s) spume droplet formation occurs, the production of coarse and supercoarse mode aerosols from wind shear atop a wave crest,^26−28^ but is not investigated here. Supercoarse mode particles experience extremely low residence times (on the order of a few seconds or less) and are not expected to influence the results below. The initiation of wave breaking is expected to cause a disruption of SML by mixing it with the underlying seawater when the surface winds exceed 8 m/s.^29,30^ Previous studies have shown that formation, thickness, and distribution of SML is strongly influenced by wind speed conditions.^31−34^ Thus, varying wind speeds can impact the SML film structure, thickness, and composition, which in turn can influence the SSA formation mechanism (i.e., largely film drops) and may change SSA composition, morphology, and mixing states.^35,36^

The effects of wind speed on composition of SSA have been studied previously. In particular, one study conducted by Gantt et al. on wind speed dependent organic mass fraction of SSA (sizes <2.5 μm) revealed a reduction in organic mass fraction with increase in surface wind speeds exceeding ∼10 m/s.^29^ The trend was attributed to a disruption of SML film structure, which results in intensive wind-driven wave breaking coupled with mixing of SML and underlying water, thus leading to the reduction of organic matter in SSA.^29^ In another study, SSA were mostly film drops (sizes <0.2 μm, predominantly organic) at wind speeds below ∼10 m/s, while SSA were largely jet drops (sizes >0.2 μm) at elevated wind speeds exceeding ∼12 m/s.^37^ Noteworthy, smaller-sized jet drops were composed of not just pure NaCl, but exhibited an appreciable amounts of organics and other inorganic components.^37,38^ These findings underscore the significant role of wind speed on composition of SSA. The chemical complexity in SSA can govern their direct and indirect aerosol effects in atmosphere.^22,39−41^ It was observed that the composition (i.e., organic and inorganic content) in SSA controls their phase state and water uptake, which alters scattering of solar radiation and their cloud condensation nuclei or ice nucleating abilities.^4,5,9,39,40^ Furthermore, prior studies performed on real SSA revealed the existence of different size-dependent morphologies (e.g., core–shell, prism-like, rounded, rod) and mixing states.^22,39,40,42−45^ Identifying morphologies and mixing states of SSA is critical to precisely predict their effects on climate as it can dictate their optical properties, water uptake and cloud condensation nuclei (CCN) ability, ice nucleating potential, and atmospheric aging.^24,46−48^ Thus, the wind speed is expected to change the composition, morphology, and mixing states of SSA, which must be comprehensively studied to accurately predict their climate-relevant effects. This is particularly significant for submicrometer SSA, due to their significant lifetime in the atmosphere relative to supermicrometer sized aerosols.^49,50^ However, no previous studies have investigated the effects of wind speed on the morphology and composition of individual submicrometer SSA as a function of size and their particle–particle variability. Such single particle measurements may be particularly important for real SSA that often display large particle-to-particle variability, as previously reported in regards to their ice nucleating potential.^9,42^

Herein, we report the effects of wind speed on the size-dependent morphology and composition of SSA. SSA were generated during a month-long mesocosm experiment, CHAOS (CHaracterizing Atmosphere Ocean parameters in SOARS, the Scripps Ocean Atmospheric Research Simulator), in summer 2022. SSA generated on the same day (August 15th) at two distinct wind speeds were compared: 10 m/s, representing a wind lull scenario and approximately reflects the global average wind speed over the ocean,^51,52^ and 19 m/s, which is characteristic of wind speeds over the Southern Ocean that are encountered during stormy conditions.^53−57^ Individual SSA (size range 0.04–1.8 μm) generated at these two wind speeds were substrate-deposited and subsequently analyzed using various complementary offline methods including filter-based thermal optical analysis, high-performance ion exchange chromatography with conductivity detection, atomic force microscopy (AFM), AFM infrared spectroscopy (AFM–IR), and scanning electron microscopy coupled with energy dispersive X-ray spectroscopy (SEM-EDX) characterization. Our findings reveal significant and size-dependent differences in the morphology and composition of nascent SSA generated at these wind speeds, highlighting the importance to consider the effects of wind speed for more accurate predictions of their climate-related impacts.

## Materials and Methods

### SSA Generation at Scripps Ocean and Atmospheric Research Simulator (SOARS)

Seawater from the Pacific Ocean at the end of the Scripps Institution of Oceanography (SIO) pier in La Jolla, CA was collected and filtered through sand filters on the pier to remove large grazers during the summer of 2022. The seawater was pumped into a 36 m x 2.5 m x 2.5 m wave channel (SOARS) in the SIO Hydraulics Laboratory to a typical channel height of 1.2 m. The SSA were generated using an air backed paddle, forming waves with an amplitude of approximately 0.5 m and peak wave frequency of approximately 15 Hz that broke independently of the wind speed within the channel. The wave packet was prescribed to have every fourth and fifth crest break, with crests terminating on a 2 m long “beach” extending from the base of the channel to above the surface of the water at a 30° angle. This beach included hard plastic netting to absorb and dissipate the wave energy, prohibiting wave reflectance. Varying wind conditions in the channel were generated through two main multiblade fans. To minimize the contribution of the surrounding air, a positive pressure in the channel was maintained through two smaller fans that introduced purified air into the system at low air flows. Wind speed measurements were collected using an anemometer (TSI 9545-A VelociCalc Air Velocity Meter), measured with a straight probe oriented perpendicular to the air flow. The wind speeds were measured at a height of 0.6 m above the water in SOARS, and extrapolated to a 10 m height value using an approach described by Hsu et al.^59^ Throughout the manuscript, the reported wind speeds correspond to these extrapolated 10 m height values. The SSA were generated on August 15th under two different wind conditions of 10 and 19 m/s. It is noteworthy that the measurements taken at 19 m/s are considered to reflect open-ocean breaking wave conditions when compared to other studies.^58,60−62^ However, due to the fixed wave amplitude in all other wind speeds measured during CHAOS, the extent of whitecap coverage cannot be compared directly to open-ocean conditions. Consequently, only the relative influence of wind speed alone can be evaluated in relation to the measurements at 10 m/s, which likely represents a wind lull scenario over a pre-existing wave field generated by higher winds. The wave field generated by the paddle is roughly equivalent to an open-ocean wave field that would be at equilibrium with a wind speed of 18.5 m/s, calculated from the average whitecap coverage following Monahan and O’Muircheartaigh.^58^ Water salinity, water temperature, and air temperature were monitored and measured as ∼32 ppt, ∼23 °C, and ∼25 °C, respectively, for both wind conditions. Before SSA generation at a particular wind speed, the wave channel headspace was run through HEPA and clean carbon 16 filters to scrub out remnant SSA and other particulates, and a sparging system used fresh water to cleanse the headspace of SSA buildup. These protocols nominally achieve a 95% efficiency of eradicating aerosols within the channel headspace relative to room air concentrations.

### SSA Collection and Size-Dependent Organic and Inorganic Mass Fraction Bulk Measurements

SSA flow generated at two wind speeds were pulled from the channel via a nozzle located ∼0.5 m preceding the beach and then collected (at ∼80 to 85% relative humidity (RH)) using a high-flow impactor (TSI model 129) at 100 L/min flow rate onto different substrates placed on three stages that have 50% cutoff aerodynamic diameter ranges of 1.0–25.0 μm, 0.25–1.0 μm, and below 0.25 μm. Prebaked 75 mm aluminum (Al) substrates were used for the two higher diameter-range stages, and prebaked 90 mm quartz fiber filters (QFF, PALL Life Sciences) were used for the smallest size range stage. All samples were stored frozen at −20 °C until analysis was conducted. No unexpected or unusually high safety hazards were encountered. Organic carbon (OC) was measured via a thermal optical analyzer (Sunset Laboratories, Forest Grove, OR), as described previously.^63^ The inorganic ions were measured via high-performance ion exchange chromatography with conductivity detection following aqueous extraction.^64^ The estimation of inorganic mass was based on the measured sodium mass which was converted to sea salt mass using a sodium/sea salt ratio of 3.26, as reported previously.^65^

### SSA Collection for Offline Single Particle Studies

SSA flow generated at two wind speeds were pulled from the channel via a nozzle located ∼0.5 m preceding the beach and substrate-deposited via a home-built silica bead dryer (ca. 50% relative humidity) using a micro-orifice uniform deposit impactor (MOUDI; MSP, Inc., model 125R) at a flow rate of 10 L/min onto different substrates including hydrophobically coated (Rain-X) silicon substrates (Ted Pella, Inc.) for AFM measurements, gold-coated silicon substrates (Ted Pella, Inc.) for AFM-IR measurements, and silicon substrates (Ted Pella, Inc.) for SEM-EDX measurements. MOUDI stages 5, 6, 7, 8, and 9 were used, corresponding to 50% cutoff aerodynamic diameter ranges of 1.00–1.80, 0.56–1.00, 0.32–0.56, 0.18–0.32, and 0.10–0.18 μm, respectively. The substrate-deposited SSA samples were stored in clean Petri dishes and kept inside a laminar flow hood (NuAire, Inc., NU-425-400) at ambient temperature (20 °C) and pressure for 2–3 months prior to single particle microscopy experiments. No unexpected or unusually high safety hazards were encountered.

### Single Particle AFM Imaging to Determine Main Morphologies and Organic Volume Fraction (OVF) of Core–Shell SSA at ∼20% RH

A molecular force probe three-dimensional (3D) AFM (Asylum Research, Santa Barbara, CA) was used for imaging individual substrate-deposited SSA at ambient temperature (20–25 °C) and pressure as described previously.^7,39,66^ A custom-made humidity cell was used to control RH within a range of ∼20 to 80%.^50^ Silicon nitride AFM tips (MikroMasch, model CSC37, typical tip radius of curvature of ∼10 nm, nominal spring constant of 1.0 N/m) were used to image individual SSA. AFM imaging was conducted in tapping mode at a scan rate of 1 Hz. Prior to AFM imaging, a hydration dehydration cycle was first carried out to limit the effect of impaction on the morphologies of deposited particles where the humidity was first increased to ∼80% RH and then after waiting at least 10 min, the RH was slowly (i.e., within several mins) decreased to ∼20% RH.^67^ The selection of these two RH values is based on the deliquescence and efflorescence RH for pure NaCl that occur at ∼75 and ∼40%, respectively.^67,68^ The AFM AC (intermittent contact) imaging mode was used to collect 3D-height and phase images of individual SSA to determine their morphology and volume-equivalent diameter, and for core–shells, quantify their organic volume fractions (OVFs) and corresponding organic coating thicknesses (OCTs), as described previously.^7,67,69^ The OVF is defined as the ratio of the shell volume to the total particle volume. Assuming the core is predominantly inorganic and shell primarily organic, the single particle OVF represents the relative amount of organic present in the particle.^7^ The OCT represents the projected thickness of organic coating around inorganic core assuming spherical particle shape.^67,70^

For morphological analysis, approximately 300 individual SSA were investigated for each wind speed (10 and 19 m/s) at four AFM-determined (at ∼20% RH) volume-equivalent diameter ranges of 0.04–0.18, 0.18–0.56, 0.56–1.00, and 1.00–1.80 μm, while for the OVF and OCT analyses, at least 20 individual SSA core–shells within each of these size ranges were investigated at both wind speeds except for the 1.00–1.80 μm size range at 10 m/s where data were based on two core–shells due to a very limited number of core–shells observed in this size range and wind speed. The relative abundance of identified morphological categories (prism-like, core–shell, rounded, rod, aggregate, and rod inclusion core–shell) and the values of the OVF and OCT were recorded as an average and one standard deviation at each size range. The observed SSA morphologies, OVF and OCT studied over the same four size ranges, were used to elucidate the effects of wind speed.

To overcome the practical limitations of studying a limited number of individual SSA using atomic force microscopy, we employed a statistical analysis to evaluate the statistical significance of our measurements. The detailed description of the approach which is based on a self-coded Monte Carlo-like simulation method can be found elsewhere.^7,71,72^ The average and one standard deviation were recorded for each morphological type of SSA at both wind speed conditions as a function of volume-equivalent diameter values. The data processing and analysis were performed by using Igor Pro (version 6.37, Wave metrics).

### Single Particle AFM-IR Measurements of SSA Composition at ∼20 to 30% RH

AFM–IR spectroscopic measurements were collected by using a commercial AFM-IR microscope (nanoIR2, Bruker) with a tunable mid-IR quantum cascade laser (QCL MIRcat-QT, Daylight solutions). Images and spectra were collected at ∼20 to 30% RH and ambient temperature (20–25 °C) and pressure on individual SSA deposited on gold-coated silicon substrates (Ted Pella, Inc.) placed on MOUDI stages 5, 6, 7, 8, and 9. Measurements were conducted using silicon nitride probes with a chromium–gold coating (Bruker, typical tip radius of curvature of ∼30 nm and a nominal spring constant range of 1–7 N/m). AFM imaging was conducted in tapping mode at a scan rate of 0.8 Hz. AFM–IR spectra were collected with a nominal spatial resolution below 35 nm and a spectral resolution of 2 cm^–1^, coaveraging over 128 laser pulses per wavenumber. A reference spectrum was taken on the substrate and subtracted from all of the corresponding spectra obtained on individual particles. Overall, ∼10 individual core–shells and ∼10 individual rounded SSA were investigated. For core–shell SSA, spectra were taken at the core and shell particle regions, while for rounded SSA, spectra were taken at an approximate center of each particle. The IR results collected were compared between the two wind speed conditions of 10 and 19 m/s.

### SEM-EDX Measurements of SSA Elemental Composition

To collect SEM-EDX data, silicon wafers (Ted Pella, Inc.) with deposited SSA were placed on a clean SEM stub and held in place by carbon tape. The data were acquired using a FEI Apreo SEM (Thermo Fisher Scientific) operating at an accelerating voltage of 10 keV and a beam current of 0.1 nA. For imaging, the immersion mode detector at short working distances (1–2 mm) was used. The standard mode detector with a working distance of ∼10 mm was employed for the EDX analysis. However, it should be noted that the analysis of samples with less than 1 μm in thickness is challenging due to the relatively low signal. To address this issue, a line scan analysis over individual particles was performed, and the beam current was adjusted to achieve acceptable X-ray counts per second (cps) within the range of 5–15 kcps. Representative particles were selected for each morphology (core–shell, rounded, rod inclusion core–shell, and aggregate) to show the variability of various elements across each representative particle.

## Results and Discussion

### Impact of Wind Speed on Size-Dependent Bulk Organic Carbon in SSA

**Figure 1:**
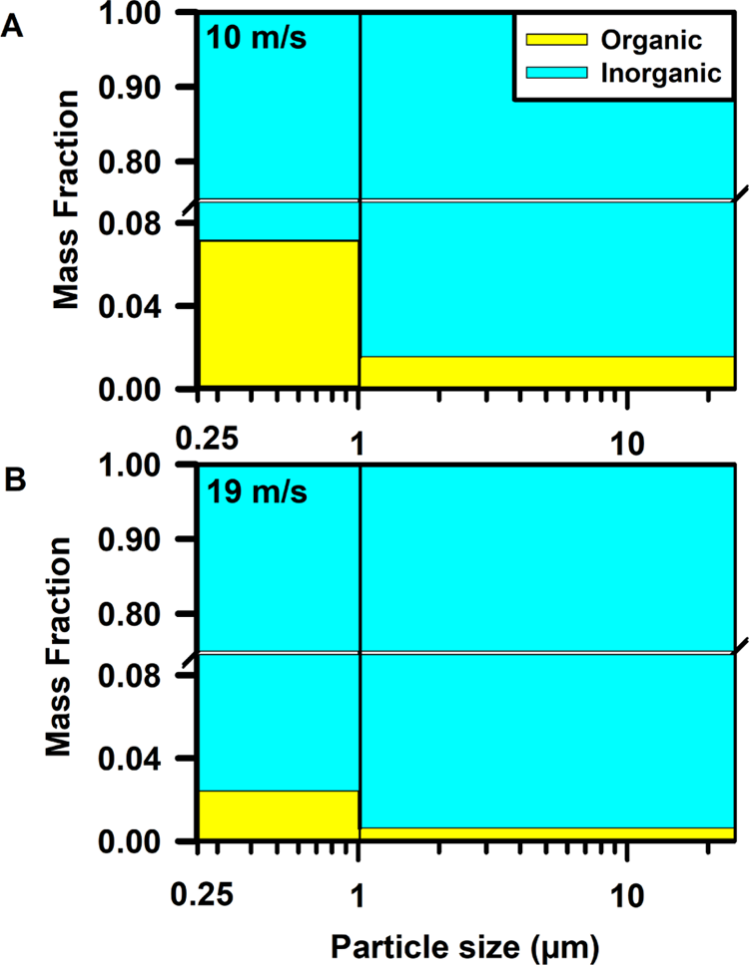
Organic and inorganic mass fractions versus particle size for SSA generated at (A) 10 m/s and (B) 19 m/s wind speeds.

Figure 1 illustrates the mass fractions of organic carbon (OC) and sea salt in SSA at two distinct wind speeds, the lowest wind speed of 10 m/s and the highest wind speed of 19 m/s, during which the experiments were conducted. In both wind speeds, mass fractions of OC in SSA increased with decreasing particle size which agree well with the prior studies.^17,73,74^ The mass fractions of OC present in both super- and submicrometer SSA under conditions of high wind speed were lower than those at low wind speed. At a wind speed of 10 m/s, the mass fraction of OC accounted for 2% within the particle size range of 1–25 μm, exhibiting an increase to 7% within the size range of 0.25–1 μm. Conversely, when the wind speed was elevated to 19 m/s, the mass fraction of OC decreased to 1% in the size range of 1–25 μm and 2% in the particle sizes ranging from 0.25 to 1 μm. This trend of decreasing OC mass fraction with increasing wind speeds is expected to result from differences in the SML film structure and composition under varying wind conditions. As proposed by Gantt et al.,^29^ the SML has an ordered film structure and organic species concentrations at low wind speeds but breaks up at higher wind speeds leading to lower organic carbon mass fraction. These variations in SML composition and film structure ultimately manifest in the composition of SSA, leading to a decrease in the mass fraction of OC in SSA at high wind speed. These findings indicate a significant effect of wind speed on the selective transfer of OC to SSA. As bulk measurements provide an ensemble-averaged perspective on the entire SSA population and do not provide an assessment on a possible particle-to-particle variability in the organic enrichment, single particle measurements were next utilized to further assess the effects of wind speed on the morphology and composition of SSA.

### Impact of Wind Speed on Size-Dependent Morphological Distribution of SSA

**Figure 2:**
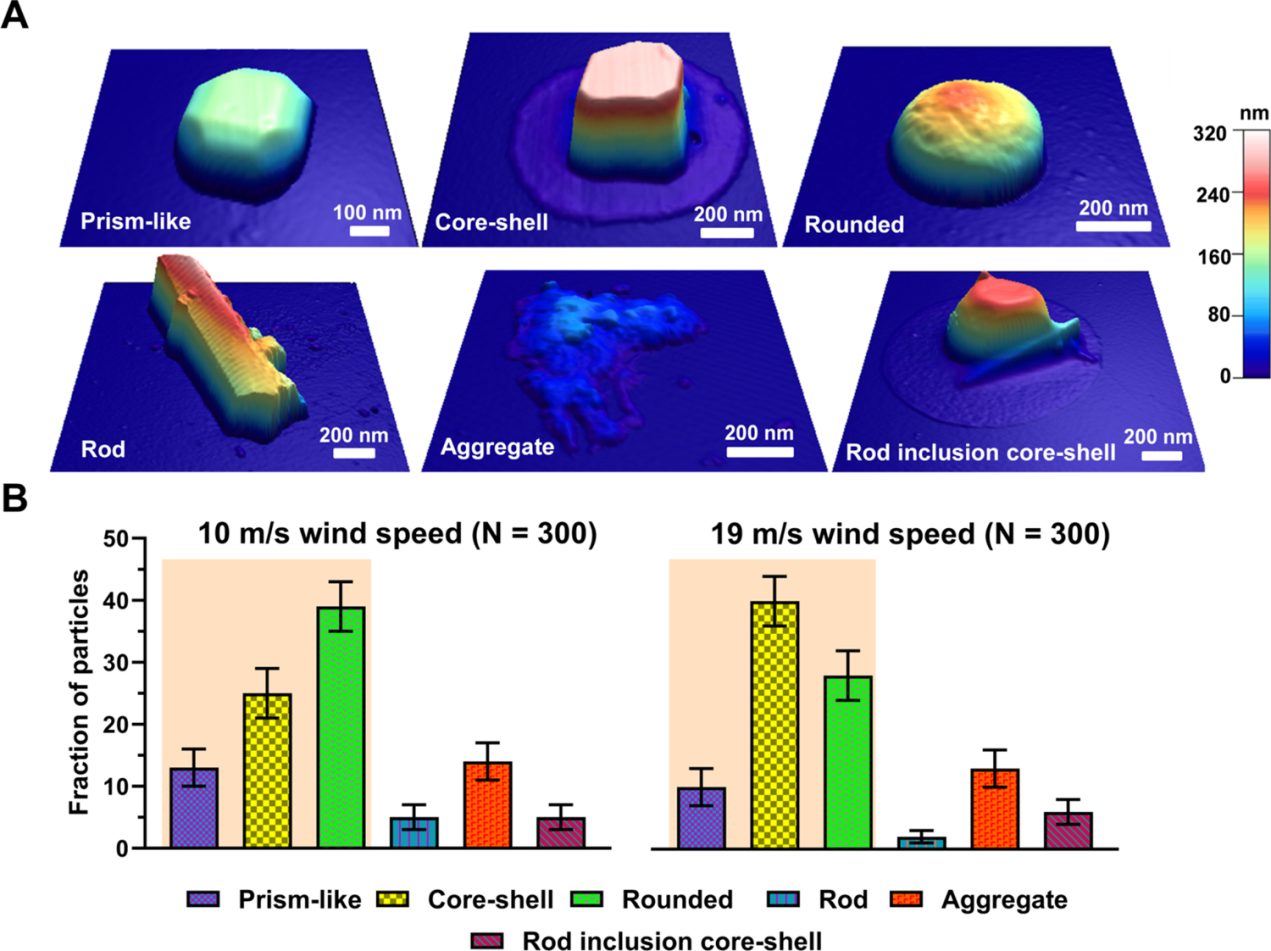
(A) Representative AFM 3D-height images at 20% RH of six main morphological categories (prism-like, core–shell, rounded, rod, aggregate, and rod inclusion core–shell) identified for SSA particles. (B) Average and one standard deviation of fraction of particles (%) from six main morphological categories at 10 m/s wind speed (total of 300 particles) and 19 m/s wind speed (total of 300 particles). For each wind speed condition, the characterized individual SSA particles had similar volume-equivalent diameter range of 0.04–1.80 μm. Statistically significant differences of three morphological categories (prism-like, core–shell and rounded) are highlighted by orange areas.

Figure 2A illustrates the representative AFM 3D-height images at ∼20% relative humidity (RH) of six main SSA morphologies (prism-like, core–shell, rounded, rod, aggregate, and rod inclusion core–shell) identified for both wind speed conditions of 10 and 19 m/s within the AFM-determined volume-equivalent diameter range of 0.04–1.8 μm.^75,76^ The classification of SSA morphologies was carried out through a qualitative analysis using AFM 3D- height and phase images as detailed in previous studies.^40,45,50,75,77,78^ Furthermore, the rod-shell morphology was also observed for both wind speed conditions (Figure S1); however, the relative abundance was less than 1% of the overall SSA population in each wind speed, thus not considered as a main morphology class. The identified morphologies of SSA are consistent with previous findings from both field observations and mesocosm experiments.^7,39,40,45,69^

Figure 2B shows the relative distribution of morphological categories at two wind speed conditions investigated over the same volume-equivalent diameter range of 0.04–1.8 μm. The relative distribution of each morphological category was determined by performing statistical distribution analysis following prior studies.^7^ From the analysis, prism-like, core–shell, and rounded morphologies collectively constitute a dominant proportion of the overall particle population. In particular, the combined fraction of prism-like, core–shell, and rounded SSA accounts for 76 and 78% at 10 and 19 m/s wind speed conditions, respectively, forming the majority of the particle population, consistent with previous mesocosm studies.^7^ Thus, the results and discussion below will largely focus on these three main morphologies (i.e., prism-like, core–shell, and rounded). The relative abundances of prism-like, core–shell, and rounded morphologies were statistically different for SSA generated at two wind speeds. Even though the aggregates had similar fractions of prism-like particles, it was not considered as a main morphology as aggregates are a combination of several morphology types with heights below 200 nm. Specifically, at 10 m/s, the majority of SSA were rounded (∼40%), while core–shells became predominant at 19 m/s (∼40%). Although the exact origin of the observed variability in the morphologies of SSA at different wind speeds remain uncertain, it likely originates from the change in SML composition and film structure due to changes in wind speed over the equilibrated wave field.^29^ Overall, these results clearly demonstrate that the change in wind speed from 10 to 19 m/s affects the relative distribution of SSA morphologies with a significant increase in core–shells at higher wind speed conditions. Next, the morphological distribution will be further assessed as a function of the particle size.

**Figure 3:**
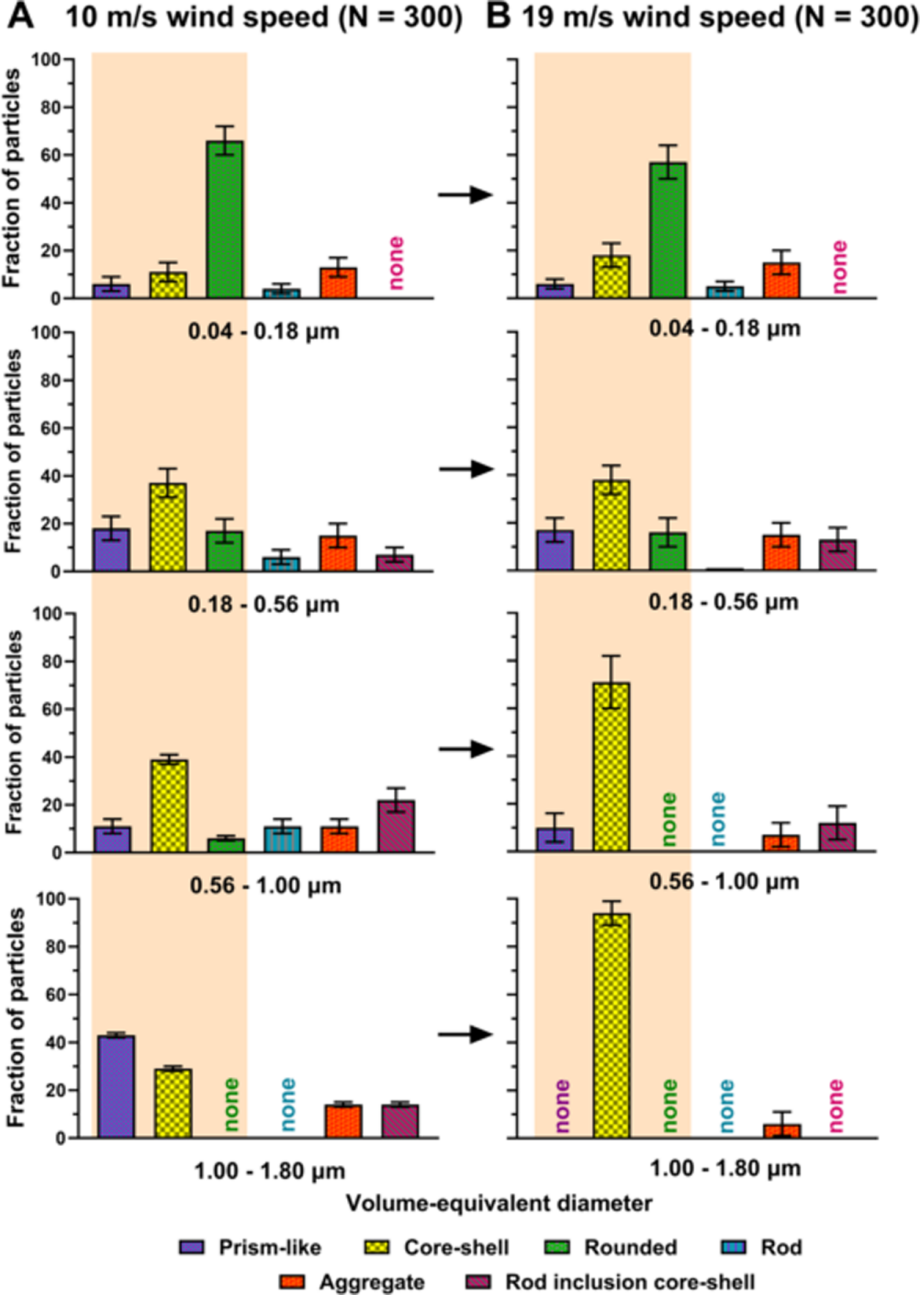
Average and one standard deviation of six main morphological categories of SSA particles (prism-like, core–shell, rounded, rod, aggregate, and rod inclusion core–shell) for four selected volume-equivalent diameter ranges of 0.04–0.18, 0.18–0.56, 0.56–1.00, and 1.00–1.80 μm generated at (A) 10 m/s and (B) 19 m/s wind speed conditions. The term “none” indicates absence of a particular morphology type within a specific SSA size range. Statistically significant differences of three morphological categories (prism-like, core–shell, and rounded) are highlighted by orange areas.

Figure 3A,B shows the morphological categorization based on the particle size within four selected volume-equivalent ranges of 0.04–0.18, 0.18–0.56, 0.56–1.00, and 1.00–1.80 μm at two different wind speeds: 10 and 19 m/s, respectively. For both wind speeds, as the particle size decreases, a significant increase in the relative abundance of rounded particles and a concurrent decrease of core–shells was observed. Additionally, for each size range, higher wind speed conditions had a larger abundance of core–shells as compared to low wind speed. Furthermore, for the wind speed of 10 m/s, a prism-like morphology was observed across all size ranges, with a larger fraction predominantly observed in the largest size range. In contrast, at 19 m/s wind speed, the abundance of prism-like particles fluctuated as a function of size without an apparent trend. The relative abundances of rod, aggregate, and rod inclusion core–shell particles were varying with respect to the particle size but without an apparent trend. We note that similar size-dependent effects were reported on SSA generated in previous wave flume studies both in the absence and presence of a phytoplankton bloom.^7,39,40,45,79^ While the observed size-dependent morphological trends are consistent with prior studies, the relative abundancies vary across different mesocosm experiments and likely originate from differences in seawater composition, biological activity, temperature conditions, and effects of wind-wave interactions affecting the SML film structure and aerosol generation mechanisms.^39,41,64,79,80^ Collectively, the observed bulk organic enrichment of smaller SSA at both wind conditions can be attributed to the increased abundance of rounded SSA, as determined by single particle imaging analysis, which, as we discuss below, are predominantly organic. Additionally, the observed reduction of the bulk organic mass fraction of SSA at elevated wind speed is likely due to the increase in core–shells that are offset by a relatively small reduction in rounded SSA.

**Figure 4:**
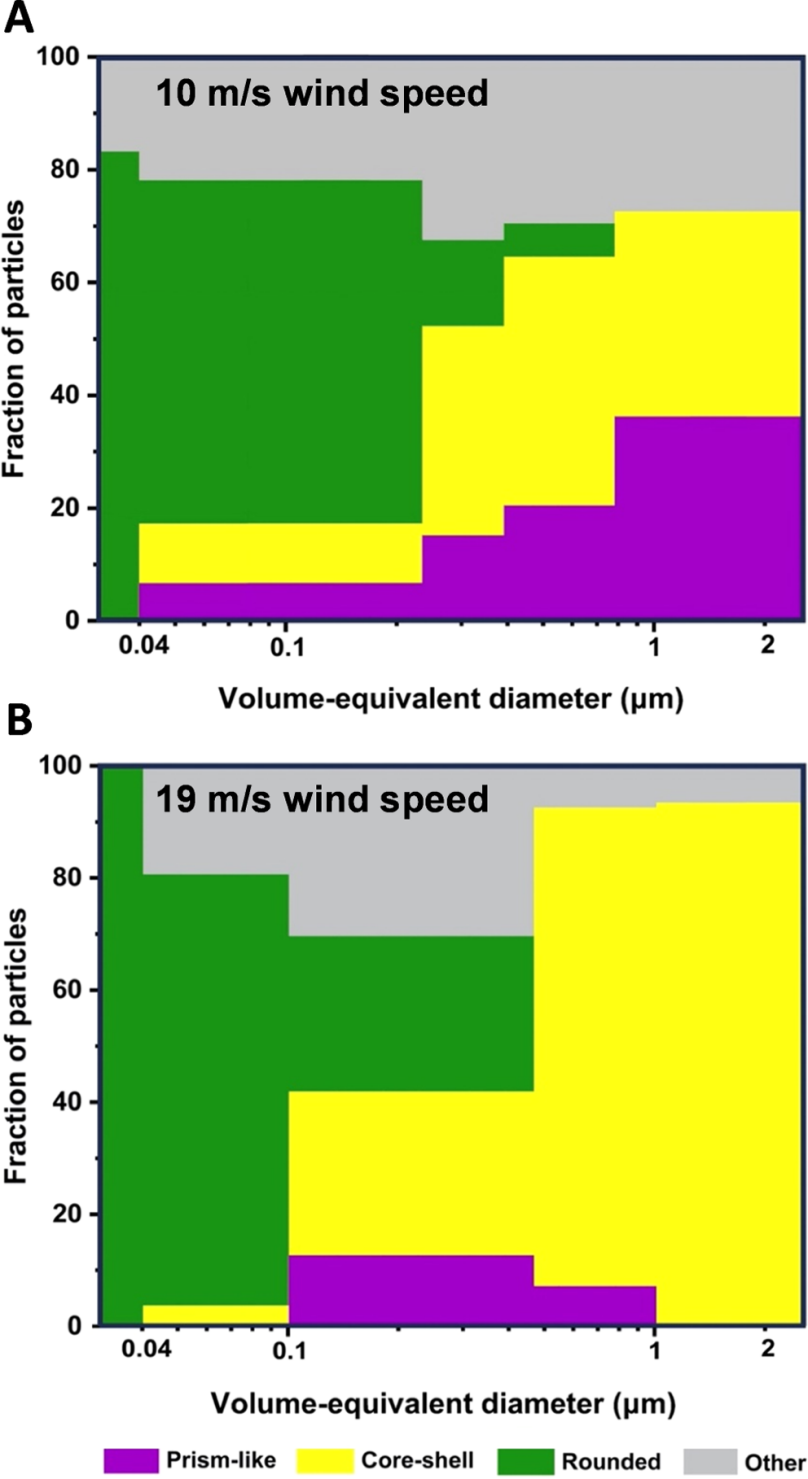
Size-resolved particle fraction distributions of dominant morphological categories of SSA particles (prism-like, core–shell, rounded, and other includes rods, aggregates, and rod inclusion core–shells) for the volume-equivalent diameter range of ∼0.04 to 2 μm generated at (A) ∼10 m/s and (B) ∼19 m/s wind speed conditions.

To better visualize the size-dependent variability of morphologies shown in Figure 3, a simplified version is presented in Figure 4 by focusing on the three main morphologies and combining the rest into the “other” category. Specifically, Figure 4A,B shows the size-resolved particle fraction distributions of main morphological categories of SSA particles (prism-like, core–shell, rounded, and “other” which includes rod, aggregate, and rod inclusion core–shell) generated at 10 and 19 m/s wind speed conditions, respectively. For the lower wind speed of 10 m/s, there was an increase in core–shell and prism-like SSA and reduction of rounded particles with the particle size increase. In contrast, at the higher wind speed of 19 m/s, there was a remarkable enhancement of core–shells with the particle size increase. This clearly indicates that there is not a single representative morphology but a dynamic and size-dependent variability in the observed SSA particles. Further studies are needed to identify the role of environmental factors in determining size-dependent particle morphology (e.g., temperature, salinity, biological activity). We anticipate such plots may be helpful to better model a representative morphology of SSA at a particular size range and under specific environmental conditions.

**Figure 5:**
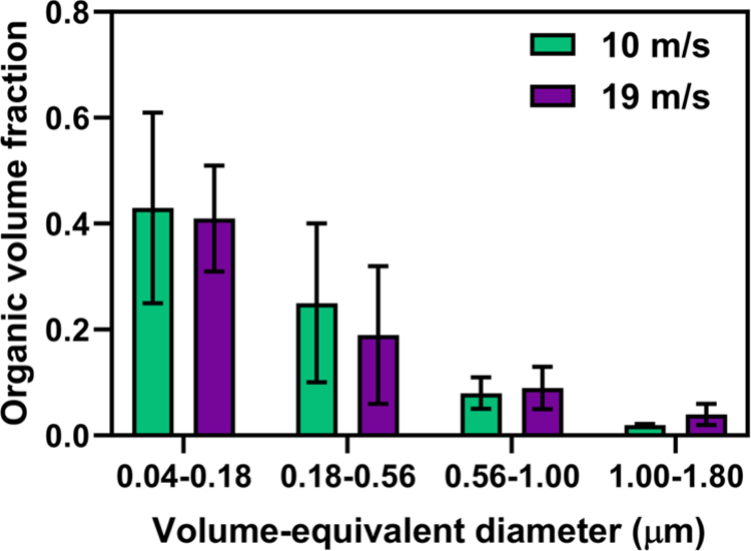
Averaged organic volume fraction measured using AFM at ∼20% RH for individual core–shell SSA particles at four selected volume-equivalent diameter ranges of 0.04–0.18, 0.18–0.56, 0.56–1.00, and 1.00–1.80 μm at 10 m/s (green) and 19 m/s (purple) wind speed conditions. Color bars and error bars represent the average and one standard deviation, respectively.

To investigate whether the relative thickness of shells of core–shells varies as a function of particle size and wind speed, the AFM-based single particle size-dependent average organic volume fraction (OVF) and corresponding organic coating thickness (OCT) of core–shell SSA at 20% RH at two wind speeds were performed with results shown in Figure 5. The OVF is defined as the ratio of the shell volume to the total particle volume and the OCT represents the projected thickness of organic coating around inorganic core assuming spherical particle shape.^7,67,70^ As will be demonstrated in the next sections using SEM-EDX and AFM-IR, the core and shell regions of core–shell SSA are predominantly inorganic and organic, respectively.^81^ Overall, as the particle size decreased, the average OVF increased for both wind speed conditions. Specifically, as the particle size decreases, the average core–shell OVF at 10 m/s wind speed increased from 0.02 ± 0.01 for the size bin 1.0–1.8 μm to 0.43 ± 0.18 for the size bin 0.04–0.18 μm, while that for 19 m/s increased from 0.04 ± 0.02 to 0.4 ± 0.1 for the same size range change. This implies organic enrichment in smaller core–shell SSA, which is consistent with prior wave flume experiments.^39^ We note that the organic enrichment in smaller core–shells is also consistent with the observed bulk organic mass fraction discussed previously. Within the reported uncertainty, there appears to be no statistically significant effect of the wind speed on the OVF of the core–shells. Furthermore, as the core–shell OCT values do not display any clear size dependency, the average value over the entire SSA studied size range of 0.04–1.8 μm was calculated. The average and one standard deviation of the OCT for 10 m/s were 10 ± 2 nm, while that for 19 m/s was 9 ± 6 nm. Overall, both the OVF and the OCT results at these two winds speeds appear to be statistically similar. Next, SEM-EDX was utilized to provide a qualitative analysis of the elemental composition of individual SSA particles under the two wind speed conditions.

### Scanning Electron Microscopy-Energy Dispersive X-ray Spectroscopy (SEM-EDX) Elemental Composition Analysis

**Figure 6:**
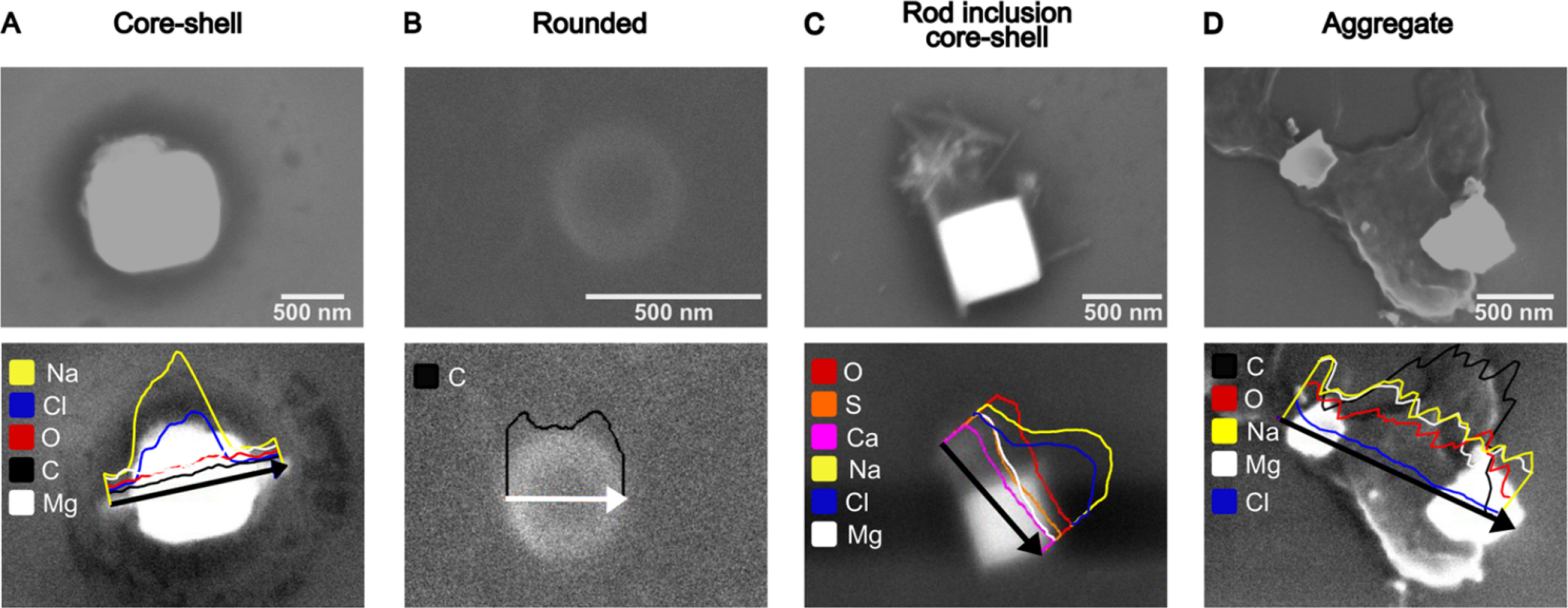
Representative SEM images (top) and EDX line scans (bottom) of various elemental compositions for (A) core–shell, (B) rounded, (C) rod inclusion core–shell, and (D) aggregate.

Figure 6 shows SEM images and EDX line scan results for representative SSA morphologies (core–shell, rounded, rod inclusion core–shell, and aggregate) to provide a qualitative assessment of the elemental composition for submicrometer SSA collected at 19 m/s wind speed (similar SEM data were observed for 10 m/s). In recent years, SEM-EDX has emerged as an important tool in offline particle analysis for characterizing the elemental composition within individual particles.^41,82^Figure 6A shows a representative core–shell with internal mixing containing a prism core and a shell of organic carbon where the core is characterized as sodium chloride (NaCl), with a Cl/Na ratio ranging between 0.4 and 0.8. This ratio is smaller than what is typically observed in seawater (1.2–1.8), suggesting a depletion of chloride ions (Cl^–^) may have occurred during and after sampling.^41,83,84^ Additionally, a slight increase in magnesium (Mg) around the core indicates a possible coating of magnesium chloride (MgCl_2_), which is consistent with previous studies conducted on SSA core–shells.^41,85,86^ Rounded particles (Figure 6B) exhibited a high carbon content, which is often associated with organic carbon. Previous studies have reported that some rounded particles possess a carbon coating with a sulfur-rich core, but these types of particles were not observed in the current analysis.^21,41,85^Figure 6C shows rod inclusion core–shells which contain a NaCl core, organic shell, and rods with elevated counts of oxygen, sulfur, calcium, and magnesium. These elements are likely associated with calcium sulfate (CaSO_4_) and/or magnesium sulfate (MgSO_4_).^21,41,85^ Last, the aggregate SSA shown in Figure 6D exhibited a diverse composition showing high counts of Na and Mg, but low counts for Cl, indicating a deficit of Cl^–^.^41,87^ The aggregate also displayed high carbon and oxygen counts, suggesting a possible complexation of Na and Mg with organic compounds.^41,88^ Overall, the SEM-EDX results show that the elemental composition of two predominant morphologies (rounded and shells of core–shell) is similar between the two wind speeds and is predominantly composed of organics. Next, the comparative organic composition of rounded and shells of core–shell morphologies at two wind speed conditions were studied using AFM-IR spectroscopy to obtain insights into the difference in organic content between these main morphologies.

### Impact of Wind Speed on Single Particle SSA Composition: Functional Group Analysis

**Figure 7:**
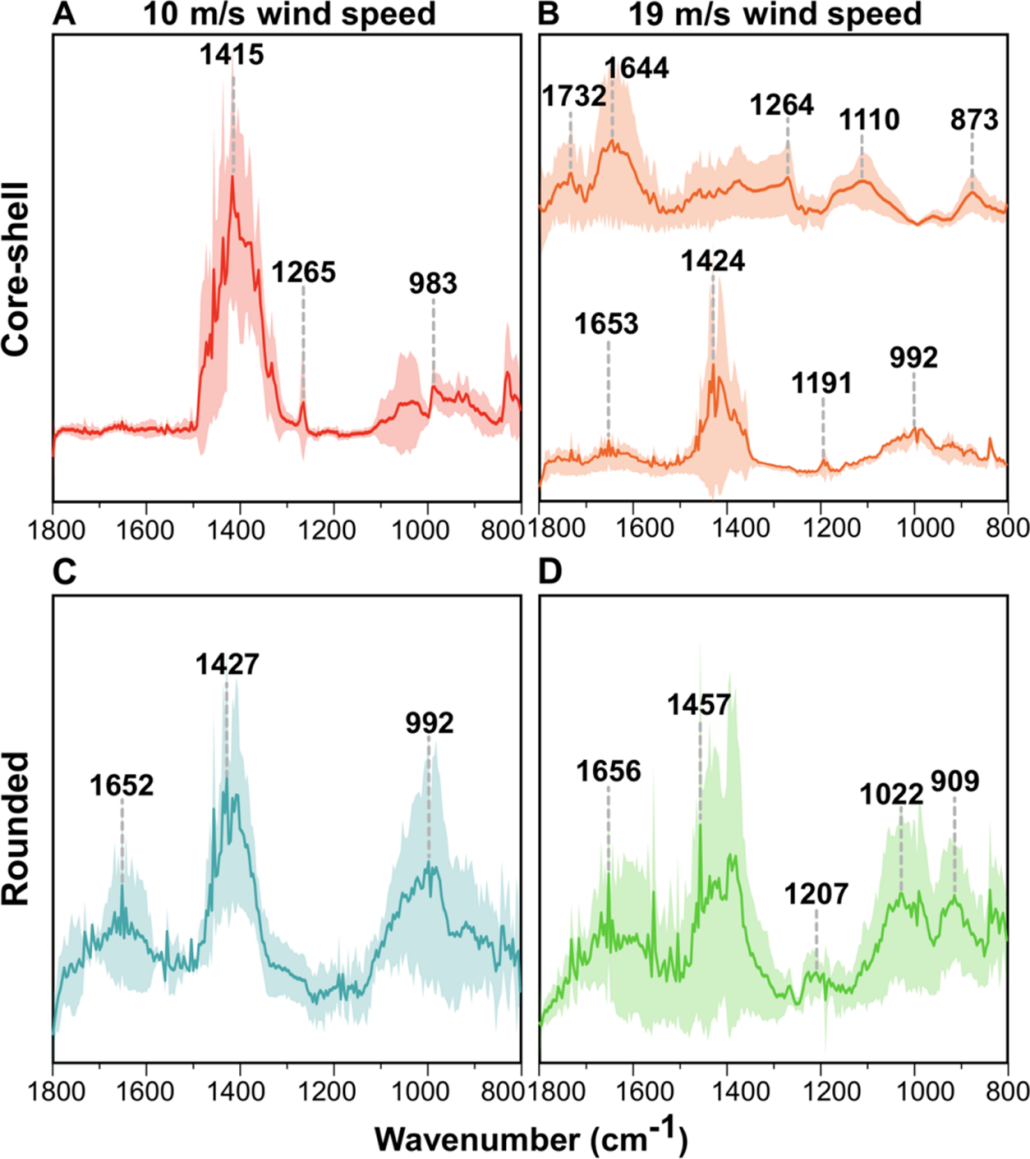
Representative AFM-IR spectra for (A) 10 m/s and (B) 19 m/s core–shell SSA, (C) 10 m/s and (D) 19 m/s rounded SSA within the volume-equivalent diameter range of 0.18–0.56 μm. Spectra were taken at shell regions for core–shell SSA and at approximately particle center for rounded SSA. The particle-to-particle variability is shown by the distinctly different spectra reported in (B) for core–shell SSA within the same volume-equivalent diameter range of 0.18–0.56 μm. Solid lines show the averaged spectra (11 individual core–shell and 7 individual rounded SSA) and shaded lines represent the 95% confidence interval.

Figure 7A,B shows the AFM-IR spectra collected at the shell region of core–shells at wind speeds of 10 and 19 m/s, respectively, with a volume-equivalent diameter ranging from 0.18 to 0.56 μm. Reference spectra for a few representative SSA-relevant compounds are provided in Figure S3. We note that the IR spectral results are not unambiguously suggesting the presence of these specific reference compounds but rather indicative of a mixture of numerous species contained within these broader encompassing classes. The core of the core–shell is largely IR inactive from 800 to 1800 cm^–1^ (likely corresponding to IR inactive compounds such as NaCl, as demonstrated using the SEM data above); thus, the spectra are not shown.^69^ The shell region spectra for both wind speeds demonstrate the presence of distinct peaks associated with functional groups that have been previously observed in SSA.^5,7,40,69,89^ The peaks in the 1550–1750 cm^–1^ region are associated with ν(C=C), ν(C=O), or ν_as_ (COO^–^); peaks between 1350 and 1470 cm^–1^ are associated with δ (CH_2_, CH_3_) or ω(CH_2_) modes. Vibrational modes in the 1000–1250 cm^–1^ region are related to ν(C–O–C), ν(C–O), or ν(C–C) stretches; the peaks between 800 and 1000 cm^–1^ are associated with ν(C–C) or (C–H) side group deformations.^7,69,84,90^

The shell spectra of core–shells at 10 m/s showed a broad peak between 1350 and 1450 cm^–1^, which corresponds to aliphatic-rich compounds (δ (CH_2_, CH_3_)), as supported by the presence of peaks in the 800–1000 cm^–1^ region (C–H wags).^84,91,92^ A similar spectrum was also observed for core–shells at a 19 m/s wind speed. However, most of the particles for this wind speed showed the presence of oxygenated functionalized groups. Specifically, the peaks in the 1640–1732 cm^–1^ region indicate the presence of carboxylic acids, esters, or carboxylates.^91^ The spectra include peaks between 1300 and 1470 cm^–1^ and a peak at 1110 cm^–1^ for δ (CH_2_, CH_3_) and ν(C–O) modes respectively,^93^ showing similar peaks as for fulvic acid (see Figure S3). Overall, at 19 m/s wind speed, most shells of core–shells displayed the presence of oxygenated organics and a small fraction of aliphatic compounds. In contrast, at 10 m/s, only aliphatic organics were observed in the shells.

Figure 7C,D shows the AFM-IR spectra collected at the approximate center of individual rounded particles at wind speeds of 10 and 19 m/s with a volume-equivalent diameter ranging from 0.18 to 0.56 μm. For both wind speeds, the spectra showed similar peaks. Peaks near 1650 cm^–1^ are associated with ν(C=O), 1350–1450 cm^–1^ corresponds to δ (CH_2_, CH_3_), and peaks between 950 and 1050 cm^–1^ show the presence of ν(C–O) stretches.^94−96^ Collectively, these results suggest that similar functional groups are present in rounded particles produced at both wind speeds, mainly composed of diverse organics including aliphatic compounds, fatty acids, and complex sugars.

Upon comparing the shells of core–shells versus rounded shells, some spectral differences can be observed for SSA generated at 10 m/s. Specifically, a peak at 1650 cm^–1^ was observed (possibly related to alcohol, saccharides, or carboxylates) (Figure S3) for rounded particles and not for shells, suggesting the presence of oxygenated compounds compared to shell region of core–shells. On the other hand, shells of core–shells and rounded SSA generated at 19 m/s wind speed showed similar peaks, suggesting similar functional group composition between shells and rounded particles at this wind speed. Particle-to-particle variability in the chemical composition is demonstrated in Figure S2, where IR spectra were taken on two different rounded particles at 10 m/s, each with the same volume-equivalent diameter range of 0.56–1.00 μm that are spectrally distinct from each other and to rounded particles shown in Figure 7. Particles in the 0.56–1.00 μm size range showed different and more diverse types of spectra than the other size ranges. Specifically, vibrational modes for inorganic compounds were observed only for rounded particles at 10 m/s which show carbonate at 1400–1500 cm^–1^ and sulfate ν_as_ (SO_4_^2–^) at 1094–1111 cm^–1^ (see Figure S2).^90,97,98^

The observed differences between core–shell SSA at 10 and 19 m/s can be explained by variations in SML film structure and in turn the mechanism of SSA formation assuming SML breakup is similar in SOARS to open-ocean conditions. First, under relatively calm conditions or low wind speeds, the SML film structure is intact and enriched with surface-active aliphatic compounds.^13,29,99,100^ As wind speed increases, typically above 8 m/s as suggested by some studies, wave breaking and increased turbulence causes the disruption of SML structure, leading to a more homogeneous water column in which the interfacial molecules contained in the SML mix with the underlying more water-soluble compounds.^29^ Consequently, the diversity of compounds that can be emitted into the atmosphere increases, with a higher proportion of water-soluble compounds being released, which are usually emitted in lower quantities under calm conditions due to the presence of the SML. Furthermore, the greater compositional diversity of core–shell submicron SSA at higher wind speeds can be attributed to the mechanism of formation. It is plausible that the film drop mechanism predominates at a wind speed of 10 m/s, whereas the jet drop mechanism prevails at 19 m/s.^18,37,101^ This finding aligns with a study demonstrating that film drops exhibit a higher fraction of aliphatic species, whereas particles generated through the jet drop mechanism contain a larger fraction of oxygen-containing compounds.^18^ While the exact origin for the observed difference in rounded and shells of core–shells at lower wind speed remains unknown, it is likely originating from a combination of several factors, including a relative contribution of jet vs film drops mechanisms to overall generation of SSA. Additionally, core–shell SSA can form when the inorganic core undergoes gas condensation and/or heterogeneous reactions at its surfaces, interacting with gaseous species during transport in the atmosphere.^102^ It has been observed that saccharides can promote and enhance the generation of core–shell SSA, whereas fatty acids have an inverse effect.^100^ The distinctive peaks corresponding to saccharides observed in core–shell SSA at a wind speed of 19 m/s are also linked to the increased proportion of core–shell SSA under these conditions. These gas phase reactions may differ for rounded particles emitted directly from the ocean without such reactions.

Table 1 summarizes key AFM-IR spectroscopic results at both wind speeds where shell of core–shell and rounded SSA are primarily enriched with organics, and specifically at 10 m/s, shell of core–shells is enriched with aliphatic compounds, and at 19 m/s, more diverse/complex organics were observed such as fatty acids, sugars, and aliphatic compounds. However, rounded particles at both wind speeds displayed similar composition with diverse organics such as aliphatic compounds, fatty acids, and complex sugars. In addition, some rounded particles at 10 m/s wind speeds showed traces of sulfate and carbonate indicating the particle-to-particle variability in composition.

**Table 1:**
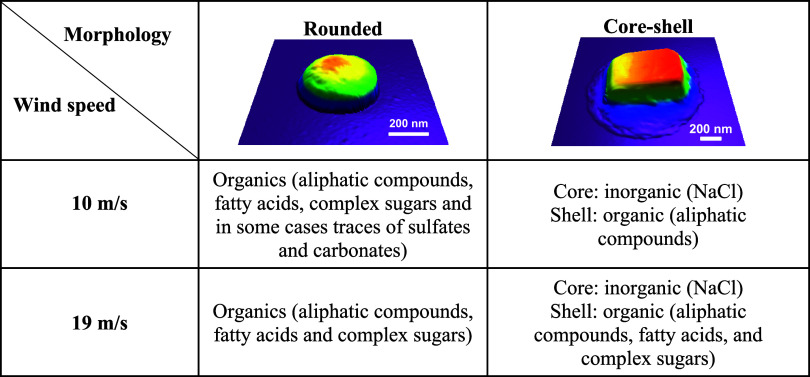
Summary of the Chemical Composition Determined by AFM-IR for the Two Main SSA Morphologies (Rounded and Core-Shell) at 10 and 19 m/s along with the Representative AFM 3D-Height Images at 20% RH

### Summary and Environmental Implications

The size-dependent morphology and composition studies of nascent SSA as a function of the size and wind speed conditions presented herein were performed for the first time. The wind speed conditions selected in this study correspond to a low wind speed of 10 m/s, which is assumed to have limited or no disruption to SML film structure, and elevated wind speed of 19 m/s, which is expected to cause a disruption of SML film structure. Our results showed clear evidence of the dynamic and size- and wind-dependent nature of the physicochemical mixing state of SSA. AFM imaging at ∼20% RH identified six main SSA morphologies present in the 0.04–1.80 μm size range at two different wind speed conditions: prism-like, core–shell, rounded, rod, aggregate, and rod inclusion core–shell where approximately 80% of SSA at both wind conditions were prism–like, core–shell, and rounded particles. Moreover, at both wind speeds, the majority of smaller SSA were rounded, while larger SSA at 10 m/s were mostly core–shell and prism-like, and in contrast, SSA at 19 m/s were predominantly core–shells for SSA with a diameter greater than 0.18 μm. As evident by filter-based measurements, both SSA at wind speeds showed an increase in the organic mass fraction with decreasing particle size. Additionally, there was a reduction in the organic mass fraction with the increase in wind speed. These results can be rationalized with complementary single particle measurements, which showed an increase in core–shell interactions that is offset by a relatively small reduction in rounded SSA. AFM–IR showed that rounded SSA at both wind speeds was largely organic with similar compositions that contained aliphatic and oxygenated species. In contrast, shells of core–shell particles showed wind-speed-dependent compositional variability, where predominantly oxygenated organics were present at higher wind speed (19 m/s), while largely aliphatic compounds were observed at lower wind speed (10 m/s). Additionally, SEM-EDX results of SSA at two wind conditions showed similar elemental profiles that were also consistent with those observed previously.^41^ The observed differences in morphology and composition of SSA at 10 and 19 m/s can be attributed to the impact of varying wind speeds on the SML film structure and composition, which in turn influences underlying mechanisms involved in the formation of SSA (i.e., film and jet drops). There could be a variability in relative contributions of these formation mechanisms toward SSA formation, which subsequently could impact the observed variability in morphologies and compositions of SSA.

These findings reveal a significant variability in SSA morphology, mixing states, and chemical composition with respect to particle size and wind speed; thus, it is expected to impact their phase state, viscosity, and water uptake, which in turn would modify the diffusion time scale of various atmospheric gases into the aerosol and therefore their atmospheric aging.^40,103^ The observed variability in SSA morphology and composition is important to consider toward accurately predicting the aerosols’ effects on the climate as they can dictate the optical properties, CCN ability, and ice nucleating potential of SSA.^24,46^ Overall, our results clearly illustrate that elevated wind speeds can result in significant changes of SSA’s physicochemical mixing state, morphological distribution, and composition. Thus, it is important to account for these size-dependent properties of SSA relating the effect of wind speed in future studies to better understand the impact of SSA on climate-relevant processes.

## Supporting Information Available

Details of AFM image of rod-shell morphology, AFM–IR spectra taken for spectrally distinct rounded particles, and AFM–IR spectra taken for laboratory deposited reference compounds (PDF)
