# Supplementary material for: Effects of Wind Speed on Size-Dependent Morphology and Composition of Sea Spray Aerosols
Source: ACS Earth Space Chem. 2024 Jul 16;8(8):1609–22. doi: 10.1021/acsearthspacechem.4c00119 (PMC11331522; doi:10.1021/acsearthspacechem.4c00119)
Supplement: Supplementary file 1 — sp4c00119_si_001.pdf [file sp4c00119_si_001.pdf]

Supporting Information

The effects of wind speed on size-dependent morphology and composition of sea spray aerosols

*Chamika K. Madawala[1], Carolina Molina [2], Deborah Kim[2], Mengnan Sun[1], Dilini Kirindigoda Gamage[1], Raymond J. Leibensperger III[3], Lincoln Mehndiratta[2], Jennie Lee[2], Chathuri P. Kaluarachchi[1], Ke'La A. Kimble[2], Greg Sandstrom[3], Charbel Harb[3], Julie Dinasquet[3], Francesca Malfatti[3,4], Kimberly A. Prather[2,3], Grant Deane[3], Dale Stokes[3], Christopher Lee[3], Jonathan. H. Slade[2], Elizabeth A. Stone[1], Vicki H. Grassian[2], Alexei V. Tivanski[1]**

[1]Department of Chemistry, University of Iowa, Iowa City, Iowa 52242, USA.

[2]Department of Chemistry and Biochemistry, University of California San Diego, La Jolla, CA 92093, USA.

[3]Scripps Institution of Oceanography, University of California San Diego, La Jolla, CA 92093, USA.

[4]Universita' degli Studi di Trieste, Department of Life Science, Trieste, 34127, Italy.

Corresponding Author

*E-mail: alexei-tivanski@uiowa.edu

CONTENT

Figure S1. Representative AFM 3D-height image of rod-shell SSA morphology at ~10 m/s wind speed within the volume-equivalent diameter range of 0.1 − 0.3 μm. The relative abundance of rod-shells at each wind speed was less than 1 % of the total studied SSA population……………………………………………………………………………S3

Figure S2. Additional IR spectra observed (at an approximate particle center) for two different rounded SSA at 10 m/s wind speed within the volume-equivalent diameter range of 0.56− 1.00 μm that were distinctly different from the average spectra reported in the main text but were not included in the main text due to them corresponding to just two particles…..…………………………………………………………………………S4

Figure S3. AFM-IR spectra of laboratory deposited reference compounds………………S5

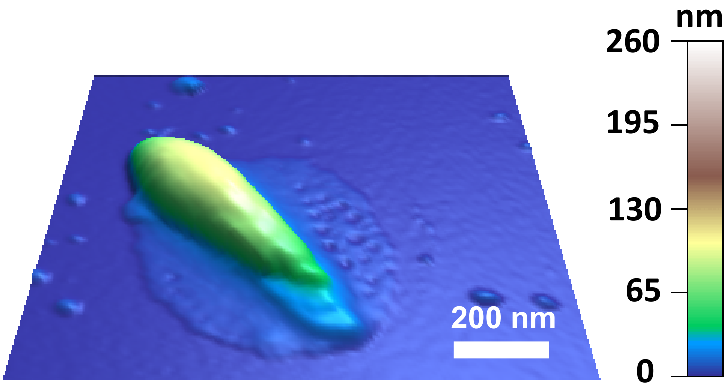

Figure S1. Representative AFM 3D-height image of rod-shell SSA morphology at ~10 m/s wind speed within the volume-equivalent diameter range of 0.1 − 0.3 µm. The relative abundance of rod-shells at each wind speed was less than 1 % of the total studied SSA population.

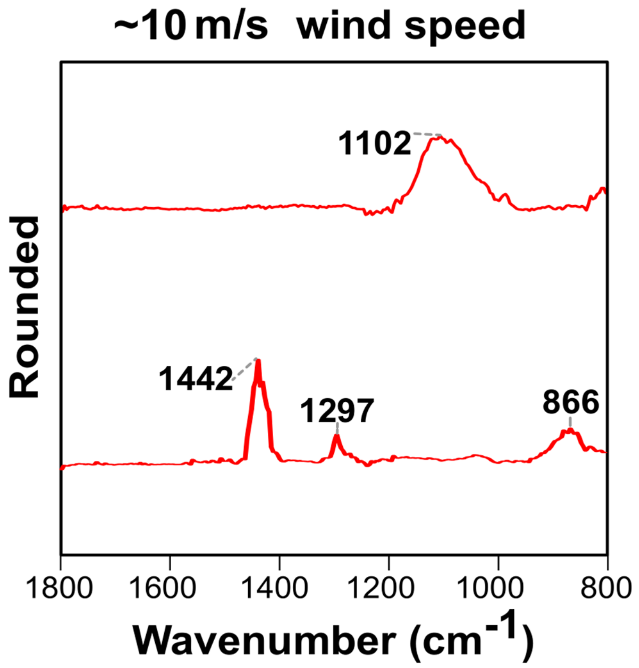

Figure S2. Additional IR spectra observed (at an approximate particle center) for two different rounded SSA at 10 m/s wind speed within the volume-equivalent diameter range of 0.56– 1.00 µm that were distinctly different from the average spectra reported in the main text but were not included in the main text due to them corresponding to just two particles.

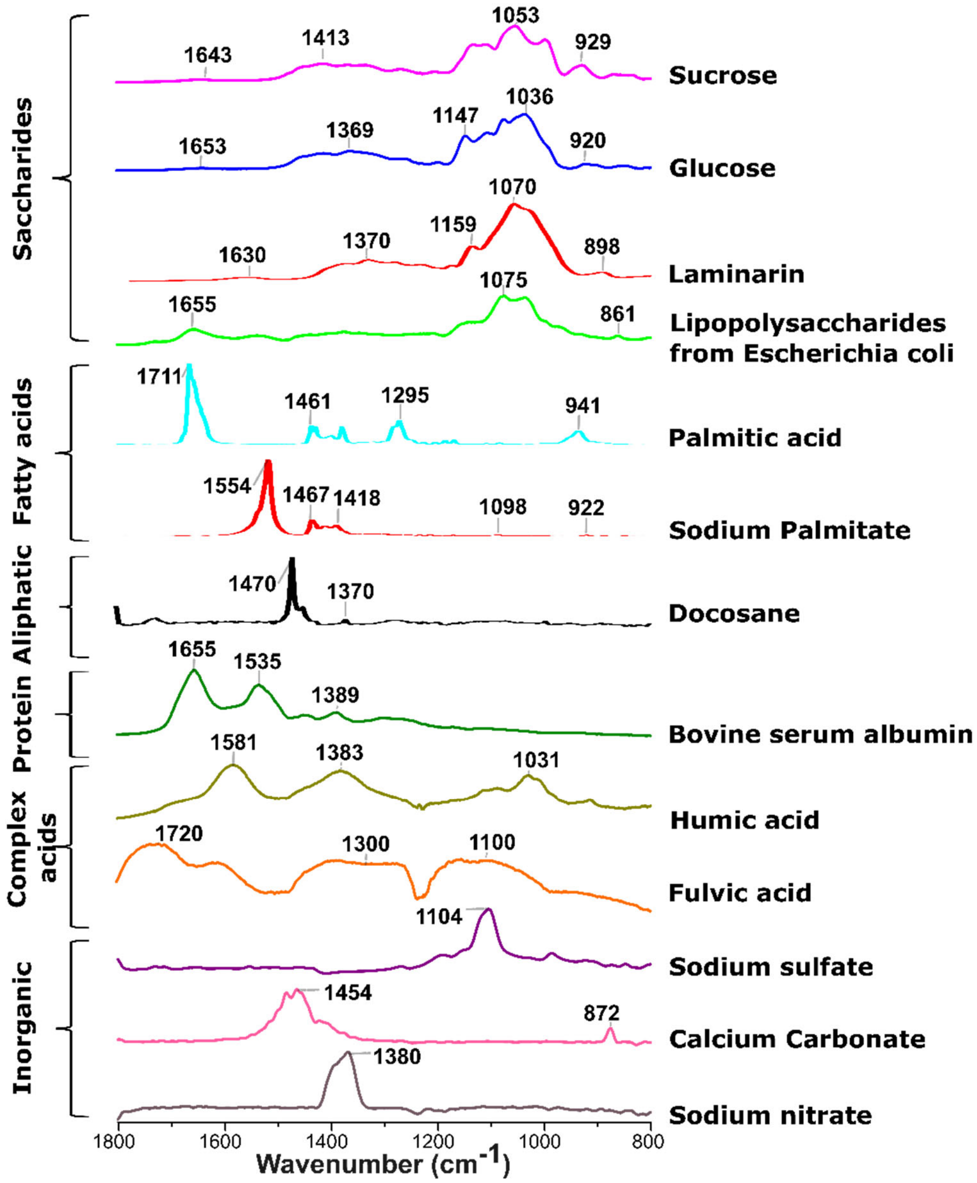

Figure S3. AFM-IR spectra of laboratory deposited reference compounds.
